# Effects of vitamin D supplementation on liver fibrogenic factors in non-alcoholic fatty liver patients with steatohepatitis: study protocol for a randomized clinical trial

**DOI:** 10.1186/s13063-019-3241-7

**Published:** 2019-03-04

**Authors:** Soraiya Ebrahimpour-Koujan, Amir Ali Sohrabpour, Farshad Foroughi, Ehsan Alvandi, Ahmad Esmaillzadeh

**Affiliations:** 1 Students’ Scientific Research Center, Tehran University of Medical Sciences, Tehran, Iran; 2 Department of Community Nutrition, School of Nutritional Sciences and Dietetics, Tehran University of Medical Sciences, Tehran, Iran; 3 The Liver, Pancreatic, and Biliary Disease Research Center, Digestive Disease Research Institute, Shariati Hospital, Tehran University of Medical Sciences, Tehran, Iran; 4 Department of Immunology, School of Medicine, Qazvin University of Medical Sciences, Qazvin, Iran; 5 Department of Cellular and Molecular Nutrition, School of Nutritional Science and Dietetics, Tehran University of Medical Sciences, Tehran, Iran; 6 Obesity and Eating Habits Research Center, Endocrinology and Metabolism Molecular -Cellular Sciences Institute, Tehran University of Medical Sciences, Tehran, Iran; 7 Department of Community Nutrition, School of Nutrition and Food Science, Isfahan University of Medical Sciences, Isfahan, Iran

**Keywords:** Trial protocol, Non-alcoholic fatty liver, Fibrosis, Vitamin D

## Abstract

**Background:**

It has been suggested that vitamin D and its receptors involve in suppressing fibrogenic signaling in non-alcoholic fatty liver disease (NAFLD). However, the effect of vitamin D supplementation on fibrogenic factors has not been investigated in NAFLD individuals with steatohepatitis. This study was designed to examine the effects on vitamin D supplementation on serum levels of vitamin D receptor (VDR), fibrogenic factors, and fibrogenic microRNAs (MiR) in NAFLD patients.

**Methods:**

Forty-six NAFLD patients will be recruited in this study. After block matching for sex and BMI, they will be randomly assigned to receive 4000 IU/day vitamin D or placebo for 12 weeks. Weight, height, and waist circumference will be measured. Determination of serum fibrogenic MiRs, laminin, collagen type IV, hyaluronic acid, vitamin D, VDR, calcium, blood glucose, serum insulin, lipid profile, liver markers (ALT, AST, total, direct, and indirect bilirubin) will be done at study baseline and at the end of the trial. Insulin resistance and insulin sensitivity will be determined using the HOMA-IR and QUICKI equation.

**Discussion:**

This is the first randomized controlled trial that will determine the effect of vitamin D supplementation on serum levels of VDR, fibrogenic factors, and fibrogenic MiRs in NAFLD patients. The results of this trial will provide clinical evidence on the effectiveness of vitamin D supplementation in controlling liver fibrosis in NAFLD patients.

**Trial registration:**

Iranian Registry of Clinical Trials, IRCT201405251485N13. Registered on 14 March 2017.

**Electronic supplementary material:**

The online version of this article (10.1186/s13063-019-3241-7) contains supplementary material, which is available to authorized users.

## Background

Non-alcoholic fatty liver disease (NAFLD) is a common progressive metabolic disorder. It starts with simple fatty liver and develops to steatohepatitis, fibrosis, and cirrhosis [1]. This clinical–pathological condition affects approximately 30% of the adult population in both developed and developing countries [2–4].

Findings from an earlier meta-analysis revealed that NAFLD patients had low levels of serum 25 (OH) D_3_. The high prevalence of vitamin D deficiency in these patients contributes to poor outcomes and progression to liver fibrosis [1, 2, 5, 6]. Experimental studies have indicated that vitamin D and its receptor (VDR) involves in suppressing fibrogenic signaling [7]. However, no evidence exists regarding the effects of vitamin D on concentrations of fibrogenic factors and liver fibrosis in NAFLD patients. Previous clinical trials have examined the effects of vitamin D supplementation on serum concentrations of inflammatory markers and lipid profiles in NAFLD patients [8–11]. Moreover, gene expression of some specific microRNAs (MiR) are disregulated in NAFLD. Most of these MiRs are related to lipid and cholesterol metabolism. There is no information about the effects of vitamin D supplementation on fibrogenic MiRs; however, some studies showed that vitamin D and VDR might modulate MiRs. As vitamin D has been shown to influence inflammation and lipid metabolism, it is hypothesized that vitamin D supplements might regulate fibrogenic MiRs in NAFLD patients. This randomized, placebo-controlled, parallel clinical trial was therefore designed to examine the effects of vitamin D supplementation on serum levels of VDR, fibrogenic factors, and fibrogenic MiRs in NAFLD patients with non-alcoholic steatohepatitis (NASH).

## Methods/design

### Participants

This parallel randomized double-blind placebo-controlled clinical trial (RCT) will be done on patients with NASH. Patients will be recruited from the Masoud Internal Specialized Clinic and Imam Khomeini Hospital, affiliated with Tehran University of Medical Sciences, Tehran, Iran. We calculated required sample size based on data from a previous study [12] by considering serum laminin as a key dependent variable, type I error of 0.05, and study power of 90%. Considering the mean difference in serum laminin levels of 136.7 ng/mL between the two groups [12], based on the suggested formula for parallel clinical trials, we reached the sample size of 18 patients in each group. Taking into account a possible drop-out rate of 30%, 23 patients will be enrolled in each group. The study is registered in the Iranian Registry of Clinical Trials website (http://www.irct.ir, identifier: IRCT201405251485N13). This study was reported based on recommended check lists for clinical trials (Additional files 1 and 2).

### Inclusion criteria

In this study, we will recruit NAFLD patients with NASH aged 20–60 years, at least six months before enrollment in the study. Diagnosis of fatty liver will be made based on findings from ultrasound reports. Patients will be recruited if they had NAFLD at grade 2 or 3. Having a vitamin D deficiency or insufficiency (serum levels of 25 (OH) vitamin D_3_ < 30 ng/mL) would be another requirement for inclusion in the study [13].

### Exclusion criteria

Individuals who are smokers, those consuming alcohol, are pregnant or lactating, or decided to get pregnant during the next three months will not be included. We also will not include individuals with some pathologic conditions affecting the liver, including viral hepatitis, any acute or chronic liver failure, liver transplantation, cholestasis, hemochromatosis, Wilson disease, α-1 anti-trypsin deficiency, suffering from diabetes, heart failure, renal failure, kidney stones, any neoplasia, inflammatory disease, usual consumption of corticosteroids, non-steroidal anti-inflammatory drugs (NSAIDs), antibiotics, proton pump inhibitors (PPIs) during the last nine months, hormone replacement therapy (HRT) or those taking high doses of estrogen, vitamin D supplements, and antioxidants during the last three months. If a participant gets pregnant during the study, consumes alcohol and/or tobacco, antioxidants, or has weight loss of > 2 kg, he or she will be excluded.

### Study design

**Fig. 1:**
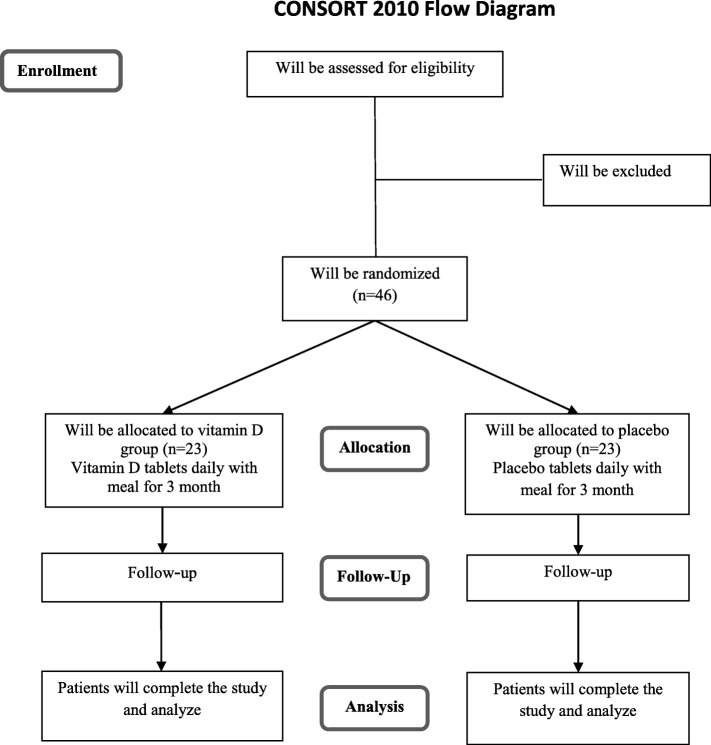
*Diagram* of the study design

This trial will be done in accordance with the guidelines laid down in the Declaration of Helsinki (1964). The study has already been approved by the bioethics committee of the Tehran University of Medical Sciences (no. IR.TUMS.VCR.REC.1395.1683). At the initial phase, we will screen 300 NAFLD patients, based on the inclusion criteria mentioned above. Those who meet the criteria will be included. All participants will be asked to complete and sign the written informed consent. Before allocating participants into the vitamin D and placebo groups, anthropometric indices, including weight, height, and waist circumference (WC), will be measured and body mass index (BMI) will be calculated. Individual questionnaires including demographic characteristics, past medical, drug and diet history, as well as socioeconomic status (SES) will be completed for each patient through the comprehensive face-to-face interview. To perform biochemical and molecular measurements, 10-mL overnight fasting venous blood samples will be obtained from each patient. All individual information of participants will be secret. A diagram of study design was shown in Fig. 1.

### Randomization

Forty-six patients will be enrolled in the whole project. We will use block randomization, in which the assignment will be done based on sex (male/female) and BMI (overweight/obese). Participants will be matched based on sex and BMI into blocks. Therefore, the block size would be four [14, 15]. This method of randomization will provide the equal number of men and women as well as overweight and obese patients that will be assigned to the intervention and placebo groups [14, 15]. Random allocation sequence will be generated using Random Allocation Software: RAS [16]. Given the total sample size of 46 and considering matched cases in terms of sex and BMI, the sample size in each subgroup of gender and BMI will be 12 ± 1. The allocation process of participants into vitamin D and placebo groups will be carried out using computer-generated random sequences by a third person who will not be involved in the project directly.

### Intervention

All patients, researchers, gastroenterologist, statistical analyst, and laboratory staff will be blind to intervention. According to the protocol of vitamin D deficiency and insufficiency treatment [13], the supplementation procedure for vitamin D will be set at a 4000 IU tablet per day for 12 weeks. The experimental group will receive 4000 IU vitamin D supplements daily with their main meals for 12 weeks. The placebo group will take placebo tablets containing lactose, which are similar to vitamin D tablets in appearance, shape, color, and smell. Participants in the placebo group will also be requested to take the tablet with their main meals for 12 weeks. The vitamin D and placebo tablets will be produced by the PARS MINOO Co., Tehran, Iran. The quality control of vitamin D tablets will be determined by the HPLC method. All patients will be blind for grouping till the end of the trial. A third person that is not involved directly in the study will package the vitamin D and placebo tablets in the bottles. The bottles will be coded as A and B and will remain unknown to researchers until the end of study. Patients will take their bottles in two time periods: at their first visit and in the middle of trial at week 6 in the laboratory of Cellular and Molecular Nutrition.

### Adherence

To determine the adherence to the intervention, individuals will be asked to record their daily consumption of supplements or placebos in a checklist given them by the investigators. To increase compliance and avoid forgetting the use of supplements and placebos, participants will receive messages on their cell phones every day from the investigators’ side. In addition, we will examine serum vitamin D levels at study baseline and at the end of the trial as a measure of compliance.

### Intervention safety

To examine the possible toxicity and hypervitaminosis that might arise from taking vitamin D supplements, serum concentrations of calcium at study baseline and end of trial will be quantified [17].

### Outcomes

**Fig. 2:**
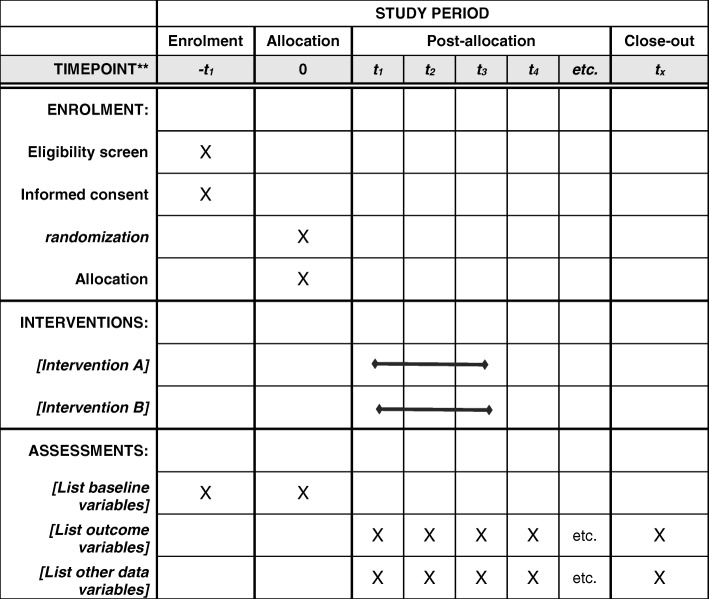
The timepoint of study period

The primary outcomes of the present clinical trial would be serum VDRs, laminin, collagen type IV, hyaluronic acid, MiR-122, MiR-21, and MiR-34a. The secondary outcomes would be liver enzymes, total, direct, and indirect bilirubin, lipid profile, and glycemic indices. We will also examine serum vitamin D and calcium concentrations. All these measurements will be assessed at study baseline and at the end of the trial. Vitamin D supplementation will be considered for the placebo group as post-trial care (Fig. 2).

### Dietary intake and physical activity assessment

To ensure lack of change in dietary intakes and physical activity of participants throughout the study, they will be asked to record their dietary intakes and physical activity in a day every two weeks. Therefore, all patients will complete six dietary records and six physical activity records (two for weekends and four for weekdays) during the study. All records would be reviewed immediately and possible problems will be resolved by interview. We will compute nutrient intake of study participants bases on the analysis of these dietary records. The software we will use for this computation is Nutritionist 4, in which the database was modified for Iranian foods. To analyze physical activity records, we will use MET-h/day values for each physical activity, based on published guidelines [18], considering the time spent by each participant .

### Anthropometric assessment

Data on anthropometric indices, including body weight, height, WC, and BMI will be obtained at study baseline and end of the trial. Body weight will be measured in a fasting state, without shoes and wearing light clothing to the nearest 0.1 kg accuracy, using a weighing calibrated scale (Seca, Hamburg, Germany). Height will be measured by mounted tape, without shoes and at a standing position near to the wall to the nearest 0.1 cm accuracy using a stadiometer (Seca, Hamburg, Germany). WC will be measured to the nearest 0.1 cm accuracy by non-stretching tape measure around the abdomen. BMI will be calculated by dividing weight in kilograms by height in meters squared.

### Blood sampling, biochemical, and molecular measurements

In a fasting state, a 10-mL venous blood sample will be taken from each patient between 07:00 and 09:00 hours. The samples will be immediately centrifuged at 20 °C, 3000 rpm for 10 min in aseptic condition. Serum samples will be separated in RNAase free micro-tubes in the clean room, where all equipment will be UV exposed for 20 min. Then the samples will be stored at − 70 °C. Serum lipid profiles (TC, HDL, LDL, TG), liver markers (ALT, AST, total, direct and indirect bilirubin), glucose, and calcium levels will be measured by the enzymatic colorimetric method using commercial kits. Serum insulin, vitamin D, VDR, laminin, collagen type IV, and hyaluronic acid will be measured using an enzyme-linked immunosorbent assay (ELISA) kit. Insulin resistance and insulin sensitivity will be determined using the HOMA-IR and QUICKI equations, respectively [19].

The gene expression of serum MiRs (MiR-122, MiR-21, and MiR-34a) will be determined by real-time polymerase chain reaction (PCR).

### Statistical analysis

Statistical analyses of all data will be performed using SPSS software version 21.0 (SPSS Inc., Chicago, IL, USA). The intention-to-treat approach will be used for data analysis. Data will be expressed as mean ± standard deviations (±SD). The one-sample Kolmogorov–Smirnov test will be used for assessing the normality of the distribution of data. In case of non-normal distribution of a variable, we will apply the log transformation. If the variables were non-normally distributed even, after log transformation, we will use non-parametric tests in the analyses. Repeated measure analysis of variance will be used to identify the effect of the intervention of outcome variables. In these analyses, baseline levels of the outcome variables will be considered as covariates. If any outcome variable was non-normally distributed, we will use the Friedman test. *P* < 0.05 will be considered statistically significant. All analyses will be done by a blind researcher before uncoding the study interventions.

## Discussion

NAFLD is one of most prevalent chronic conditions worldwide [20–22]. Its spectrum includes hepatic steatosis, NASH, and chronic fibrosis and liver cirrhosis [23, 24]. Vitamin D deficiency is highly prevalent among NAFLD patients and this deficiency is related to the degree of liver function and degree of fibrosis. It has been suggested that vitamin D deficiency could be used as a prognostic index and a diagnostic tool in NASH [25]. Previous investigations have indicated that vitamin D and its receptor VDR were involved in the inhibition of fibrogenic pathways and gene expression of fibrogenic factors in the liver [7].

To the best of our knowledge, this is the first RCT that will determine the effect of vitamin D supplementation on serum levels of VDR, fibrogenic factors, and fibrogenic MiRs in NASH patients. The block randomization method of study participants is one of the strengths of the present clinical trial. The results of this trial will provide clinical evidence on the effectiveness of vitamin D supplementation in controlling liver fibrosis in NAFLD patients with NASH.

## Trial status

The authors confirmed that this is the first version of the protocol. Recruitment began on 15 April 2018 is expected to be completed by 15 January 2019.

## Additional files

Additional file 1: Supplementary file 1: The SPIRIT 2013 Checklist. (DOC 120 kb)

Additional file 2: Supplementary file 2: The CONSORT 2010 Checklist. (DOC 217 kb)

## Abbreviations

ALT: Alanine aminotransferase
AST: Aspartate aminotransferase
BMI: Body mass index
ELISA: Enzyme-linked immunosorbent assay
HDL: High-density lipoprotein
HOMA-IR: Homeostatic Model Assessment for Insulin Resistance
HRT: Hormone replacement therapy
LDL: Low-density lipoprotein
METs: Metabolic equivalents
MiR: MicroRNA
NAFLD: Non-alcoholic fatty liver disease
NASH: Non-alcoholic steatohepatitis
NSAIDs: Non-steroidal anti-inflammatory drug
PPIs: Proton pump inhibitors
QUICKI: Quantitative Insulin Sensitivity Check Index
RCT: Randomized clinical trial
SD: Standard deviation
SES: Socioeconomic status
TC: Total cholesterol
TG: Triglyceride
UV: Ultraviolet
VDR: Vitamin D receptor
WC: Waist circumference
